# Deficient neutrophil responses early in influenza infection promote viral replication and pulmonary inflammation

**DOI:** 10.1371/journal.ppat.1012449

**Published:** 2025-01-17

**Authors:** Henry H. Gong, Matthew J. Worley, Kyle A. Carver, Caleb J. Godin, Jane C. Deng

**Affiliations:** 1 Graduate Program in Immunology, Ann Arbor, Michigan, United States of America; 2 Research Service, VA Ann Arbor Healthcare System, Department of Veterans Affairs Health System, Ann Arbor, Michigan, United States of America; 3 Division of Pulmonary and Critical Care Medicine, Department of Internal Medicine, University of Michigan, Ann Arbor, Michigan, United States of America; 4 Medicine Service, VA Ann Arbor Healthcare System, Department of Veterans Affairs Health System, Ann Arbor, Michigan, United States of America; University of Zurich: Universitat Zurich, SWITZERLAND

## Abstract

Neutrophils play key protective roles in influenza infections, yet excessive neutrophilic inflammation is a hallmark of acute lung injury during severe infections. Phenotypic heterogeneity is increasingly recognized in neutrophil populations; however, how functional variation in neutrophils between individuals determine the diverse outcomes of influenza remains unclear. To examine immunologic responses that may drive varying outcomes in influenza, we infected C57BL/6 (B6) and A/J mice with mouse-adapted influenza A virus A/PR/8/34 H1N1. A self-resolving dose in B6 mice was lethal in A/J mice, which had increased viral load throughout infection accompanied by prominent bronchoalveolar neutrophilia and pulmonary vascular leakage preceding mortality. Notably, the B6 mice heavily recruited neutrophils to lungs early in infection while A/J mice failed to do so. Neutrophils from A/J mice additionally displayed reduced neutrophil extracellular trap (NET) release and reactive oxygen species (ROS) generation compared to B6 mice early in infection, suggesting the failure to control virus in A/J mice was a product of deficient neutrophil response. To determine if variation in neutrophils between strains governed viral control and inflammation, we adoptively transferred bone marrow neutrophils from B6 or A/J donors to A/J recipients early in infection and found that the transfer of B6 neutrophils enhanced viral clearance and abrogated the dissemination of CXCL1 and IL-6. The transfer of A/J neutrophils, however, failed to achieve either. Furthermore, B6 neutrophils were capable of greater levels of viral killing *in vitro* than their A/J counterparts. These results suggest that a key moderator of inflammation in influenza infection is the control of virus by neutrophils early in infection. Thus, host-specific differences in both the recruitment of these cells as well as interindividual variation in neutrophil ability to support viral clearance may in part dictate differing susceptibility to respiratory viral infections.

## Author summary

The role of neutrophils in influenza is unclear. Although they are associated with increased severity of infection and can cause host tissue damage, their absence reduces survival in mouse studies of severe influenza infection. To study how neutrophils may affect the severity of infection, we have infected the C57BL/6J (B6) and A/J strains of mice with H1N1 influenza A virus. The A/J mice are highly susceptible to infection compared to B6 mice, allowing us to compare aspects of the immune response that may mediate this vulnerability. Compared to the B6 mice, we found the A/J mice had high viral loads, disseminated inflammation, and higher mortality. A main difference in the immune response appeared to be deficient early recruitment of neutrophils to lungs of A/J mice. When we transferred B6 neutrophils into infected A/J lungs early in infection, we found viral loads were reduced and inflammation was restricted to the lungs. A/J neutrophils failed to confer these benefits, however, and performed worse than B6 neutrophils in viral killing assay. Our study indicates that an effective early neutrophil response is highly beneficial during influenza infection and that differences in neutrophil function between individuals may underlie susceptibility to infection.

## Introduction

Influenza A virus (IAV) infections remain a major threat to global health having surpassed pre-COVID-19 levels by the end of 2022. Annually, around 300,000 people succumb to the disease globally, while the last century has demonstrated the potential for repeated widespread surges as exemplified by the 1957, 1968, and 2009 flu pandemics [1,2]. Severe IAV infections often develop into acute respiratory distress syndrome (ARDS) characterized by barrier breakdown in the alveoli and severe lung injury, which may be caused directly by viral infection and damage to the respiratory epithelium [3]. Just as prominently, however, an overexuberant and neutrophil-dominant immune response is known to lead to lethal immunopathology. Excessive inflammation from neutrophil processes including degranulation, neutrophil extracellular trap (NET) release, reactive oxygen species (ROS) generation, and inflammatory cytokine production heavily contribute to lung pathology during severe influenza infection and underlie susceptibility in older individuals [4–6]. Conversely, neutrophil depletion studies in mice have indicated that neutrophils serve a critical role in limiting early viral replication and ensure survival during moderately severe infections or prolong survival against lethal infections, underscoring the need to understand whether specific components of neutrophil responses that govern viral clearance can be uncoupled from those that mediate lung injury [7–11]. In contrast to early depletion, however, neutrophil depletion late in infection appears to be harmless or even beneficial [4,12]. Furthermore, the use of a low-dose anti-Ly6G regime to attenuate neutrophil numbers has been shown to enhance survival during lethal influenza infection, suggesting that neutrophils may become deleterious only with an excessive inflammatory response [10]. In support of this notion, recent work has indicated that inflammasome activation may underlie deleterious neutrophil functions with Gasdermin D (GSDMD) knockout mice protected from influenza-induced mortality despite similar viral loads and lung neutrophil recruitment compared to wild-type [12]. Thus, rather than being strictly harmful, the contributions of neutrophils in influenza appear to vary depending on the timing of their response, the scale of their recruitment, and their activation profile. Neutrophil contributions to injury and resolution during influenza infections appear to be highly context dependent and require further study to fully understand.

It is increasingly recognized that neutrophils are much more plastic and multifunctional than previously appreciated with functionally heterogeneous subsets rising in steady state and in disease [reviewed in [13,14]]. While scRNA-seq analysis has shown influenza infection invokes distinct subsets of neutrophils in humans, it remains unclear if specific neutrophil phenotypes may predispose individuals to severe outcomes in influenza [15]. Bulk RNA-seq datasets have stratified peripheral leukocyte gene expression in accordance with influenza infection severity, generally showing a gradient from gene signatures dominated by interferon-related responses in mild infections towards neutrophil-dominated inflammatory signatures in severe infections [16–20]. Notably, the analysis by Zerbib *et al*. implicated the neutrophil-specific genes *OLFM4* and *CD177* as the top 2 most expressed genes that differentiated moderate and severe influenza [19]. The proteins for these genes are expressed only in phenotypically distinct subsets of neutrophils that are variably present in healthy individuals [21,22]. This data suggests that interindividual variation in neutrophil functions and subset composition may therefore bias the severity of influenza infections.

To examine whether interindividual neutrophil differences may drive susceptibility to severe influenza infections, we used the mouse-adapted A/PR/8/34 H1N1 (PR8) IAV infection model in two inbred laboratory mouse strains, the C57B6/LJ (B6) and A/J strains. A/J mice have markedly higher susceptibility to influenza and present with high viral load, granulocytic lung infiltration, and cytokine overexpression relative to B6 mice [23–25]. Susceptibility to influenza in A/J mice appears to be driven by defects in the innate immune response, as they are protected from secondary influenza infection [26]. We hypothesized that host-specific differences in neutrophil phenotypes may underlie the strain-related differences in susceptibility to influenza. During influenza, we found that A/J mice had a delayed neutrophil response which led to increased viral replication and exacerbated inflammation. The intranasal adoptive transfer of B6 neutrophils to infected A/J mice early in infection greatly enhanced viral clearance and prevented the dissemination of inflammation. The transfer of A/J neutrophils, however, failed to mediate these effects. Finally, we found B6 neutrophils had enhanced virucidal capability *in vitro* compared to A/J neutrophils.

## Results

### A/J mice have high susceptibility to influenza infection

**Fig 1:**
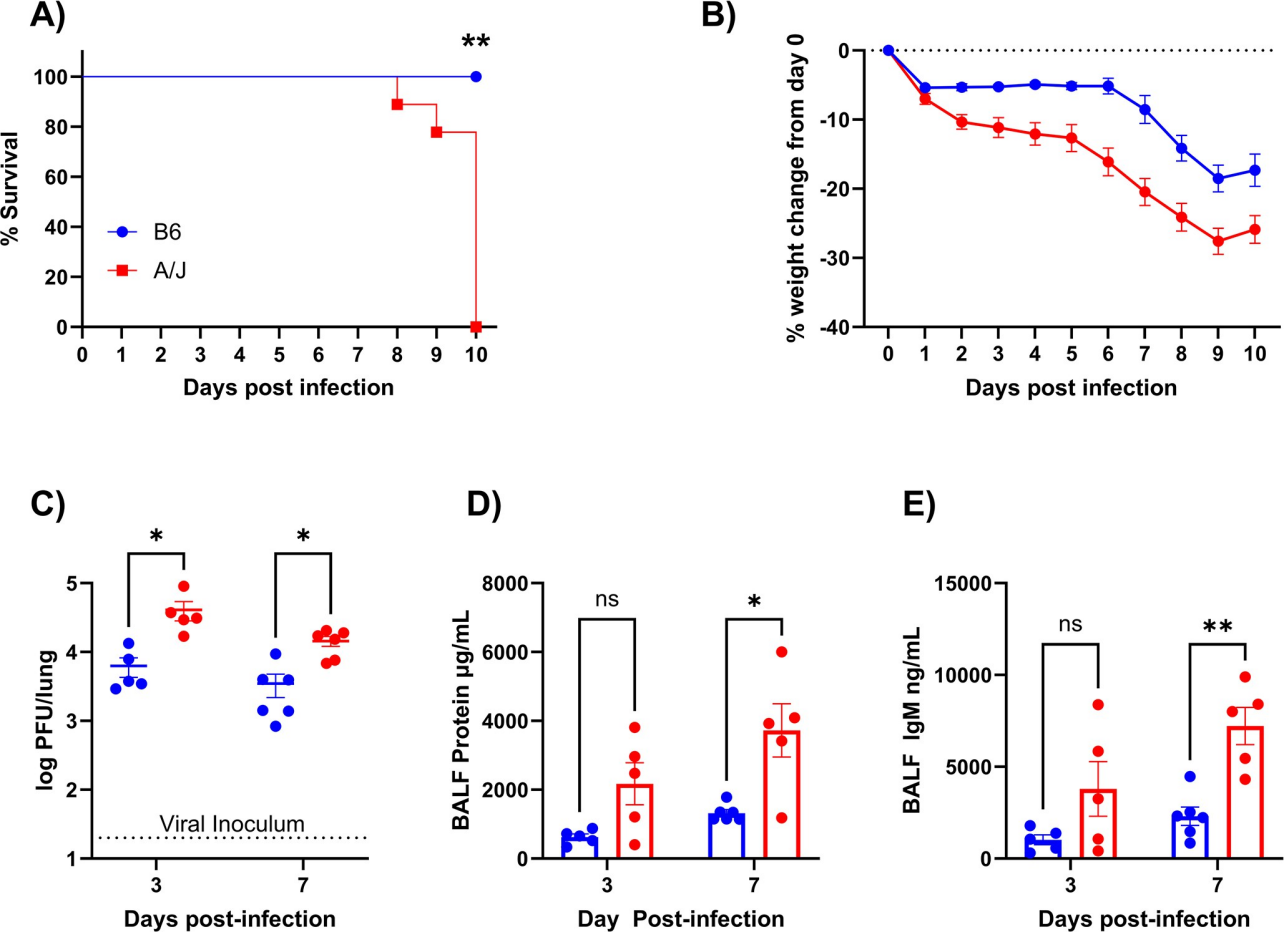
A/J mice are far more susceptible to influenza infection than B6 mice. A/J and B6 mice were infected intratracheally with 20 PFU of PR8 IAV. (A) Survival curve for B6 and A/J mice. (B) Weight loss curve shown as % change from infection day 0. (C) Lung viral load was measured from lung homogenates at day 3 and day 7 post-infection using TCID50 assays. (D) Total protein was measured in BALF with BCA assay as a measure of inflammation and lung leakage at day 3 and 7. (E) BALF was measured for IgM as a marker of vascular leakage through the alveolar epithelium at day 3 and 7 (For A and B: n = 10 B6, 15 A/J; C-E: n = 10-11/group). Data presented are representative of 2 experimental repeats. Statistical analysis by Log-rank test for survival or with Mann-Whitney U test with two-stage step-up method for multiple comparisons correction, false discovery rate (FDR) = 0.05, where applicable. Significant differences are denoted * p<0.05, ** p<0.01.

Using the A/J and B6 strains of mice, we sought to characterize determinants of susceptibility to influenza. We found that 20 plaque-forming units (PFU) IAV delivered intratracheally was 100% lethal to A/J mice by day 11 post-infection (PI), with the earliest death occurring on day 8 PI (Fig 1A). A/J mice began losing weight by day 2 and continued to decline in weight and clinical condition until euthanasia was required. In contrast, however, weight loss in B6 mice was delayed until day 7 and recovered after day 9, contrasting with the more rapid and extensive weight loss found during infection of A/J mice. (Fig 1B).

We measured viral burden in the lung tissue on day 3 PI, as an early time point when A/J mice had diverged from B6 mice in terms of weight loss, and on day 7 PI, a critical time point that precedes mortality in A/J mice and coincides with weight loss in B6 mice. At both timepoints, viral burden was significantly higher in A/J lungs than in B6 lungs (Fig 1C). Viral pneumonia leading to ARDS is the main cause of death in influenza infections and is marked by alveolar barrier hyperpermeability [27]. To assess lung vascular and epithelial barrier breakdown, we measured total protein and IgM levels from bronchoalveolar lavage fluid (BALF) at days 3 and 7 PI. Total protein levels were higher in A/J BALF compared to B6 BALF at day 7 but not at day 3, suggesting increased bronchoalveolar inflammation and leakage at the later time point (Fig 1D). Accordingly, we also found that BALF IgM levels were significantly higher in A/J mice at day 7 but not at day 3 PI, indicating increased pulmonary endothelial-epithelial barrier permeability late in infection (Fig 1E). Notably, we did not find evidence of increased pulmonary barrier breakdown at day 3 in A/J mice despite their higher viral loads at that time point. As an overexuberant immune response is known to be another major driver of lung damage in influenza infection, we next assessed lung leukocyte recruitment during infection.

### A/J mice have deficient neutrophil recruitment early in infection relative to B6 mice

**Fig 2:**
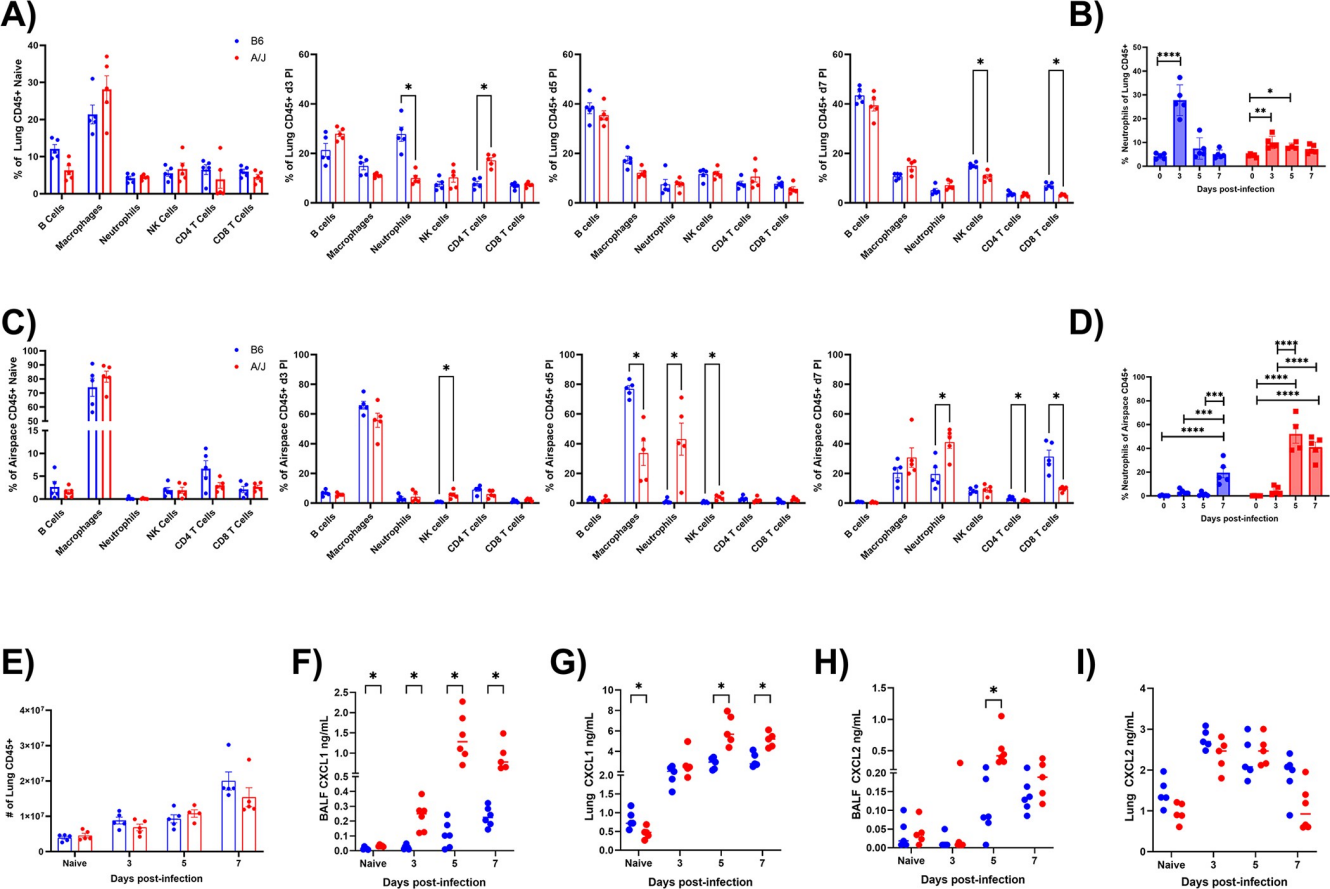
A/J mice exhibit delayed neutrophil recruitment kinetics and distinct neutrophil compartmentalization compared to B6 mice. Bronchoalveolar lavage was performed on lungs followed by enzymatic digest following infection at the specified time points for cell differentials. Proteins were detected from whole lung homogenates or from 1^st^ mL of BALF recovered. (A) Lung leukocyte differentials from uninfected B6 and A/J mice at days 3, 5, and 7 PI. (B) Lung neutrophil proportions were compared between time points within each strain. (C) Bronchoalveolar leukocyte differentials from naïve B6 and A/J mice at days 3, 5, and 7 PI. (D) Bronchoalveolar neutrophil proportions were compared between time points within each strain. (E) Total lung leukocyte counts in uninfected mice and at days 3, 5, and 7 PI. (F) BALF CXCL1 levels in naïve mice and at days 3, 5, and 7 PI. G) Lung tissue CXCL1 levels in naïve mice and at days 3, 5, and 7 PI. (H) BALF CXCL2 levels in naïve mice and at days 3, 5, and 7 PI. (I) Lung tissue CXCL2 levels in naïve mice and at days 3, 5, and 7 PI (n = 5-10/group). Data presented are representative of 2 experimental repeats. Statistical analysis performed using Mann-Whitney U test with two-stage step-up method for multiple comparisons correction, false discovery rate (FDR) = 0.05, where applicable or with one-way ANOVA using Tukey’s post-hoc test. Significant differences are denoted * p<0.05, ** p<0.01, *** p<0.001, **** p<0.0001.

Having found that A/J mice had increased viral loads throughout the infection but increased alveolar leakage only late in infection, we assessed immune cell recruitment in uninfected mice and at days 3, 5, and 7 PI in the lungs and the bronchoalveolar space. We found no difference in lung leukocyte differentials in uninfected mice. We found that B6 mice had a significantly higher presence of neutrophils in the lung tissue at day 3 compared to A/J mice, comprising nearly 30% of all leukocytes present in B6 compared to about 10% in A/J mice. In addition, CD4+ T cells were a larger portion of cells in A/J lungs at this time point (Fig 2A). By day 5, however, the proportion of neutrophils in B6 mouse lungs had declined to under 8% of leukocytes and remained low through day 7, when more NK cells and CD8+ T cells appeared in B6 lungs relative to A/J lungs (Fig 2A). The proportion of neutrophils in A/J mouse lung tissue increased following infection but remained below 10% of leukocytes throughout infection and declined to pre-infection levels by day 7 PI (Fig 2B). In the airspaces (i.e., bronchoalveolar lavage samples), neutrophils were almost undetectable in uninfected mice and present as a minor population (<5%) at day 3 PI in both strains of mice (Fig 2C). By day 5 and through day 7 PI, however, at least 40% of leukocytes in the BALF of A/J mice were neutrophils and were found at significantly higher proportions compared to B6 mice. At day 5 PI, macrophages were decreased, and NK cells were slightly increased in A/J airspaces compared to B6 animals. At day 7 PI, the proportions of CD8+ T cells and CD4+ T cells were enhanced in B6 airspaces compared to A/J airspaces (Fig 2C). From day 5 to day 7 PI, neutrophil numbers greatly increased in B6 airspaces but their proportions remained significantly lower compared to A/J airspaces nonetheless (Fig 2C, D). Despite the higher viral loads in A/J mice, total lung leukocyte counts remained equivalent between strains at all time points assessed (Fig 2E).

The CXCR2 ligands CXCL1 and CXCL2 are known to be two of the most common and potent chemokines that regulate neutrophil recruitment [28]. We measured their concentrations in the BALF and in lung homogenates from uninfected mice and at days 3, 5, and 7 PI to identify possible mediators in the disparate kinetics and localization of neutrophils in these two strains of mice during infection. No differences were detected for these chemokines in uninfected mice (Fig 2F–2I). CXCL1 was elevated in the BALF at all post-infection timepoints in A/J mice and reached a peak average of 1385pg/mL at day 5 PI, nearly 11-fold greater than the levels of their B6 counterparts at that time point (Fig 2F). At day 7 PI the BALF CXCL1 concentration of the A/J mice were still over 4-fold greater than the concentrations in B6 mice, which had reached their peak values. Similarly, in the lung tissue, CXCL1 was elevated at day 5 and 7 in A/J mice compared to B6 mice although to a lesser degree (day 5: 2.16-fold, day 7: 1.62-fold) than in the BALF (Fig 2G). CXCL2, on the other hand, was elevated in the BALF only at day 5 in A/J mice relative to their B6 counterparts and was not different in the lungs at any time point. (Fig 2H, I). We additionally measured levels of CXCL5, another major ligand for CXCR2, as well as levels of CCL2, CCL3, CCL4, and CCL5, which are ligands of the receptors CCR1, CCR2, and CCR5 that are expressed by neutrophils under inflammatory conditions. Interestingly, we found CXCL5 was elevated in the lungs and BALF of A/J mice at all infection time points as well as in the BALF of uninfected A/J mice. We found CCL2, CCL3, and CCL4 were increased in the A/J lungs at days 3 and 5 PI. We also observed that CCL3, CCL4, and CCL5 were elevated in the BALF of A/J mice at d5 PI. CCL2 also elevated at day 3 PI and CCL3 was also elevated at day 7 PI in the BALF (S1 Fig). Beginning at day 3 PI, multiple chemokines contribute to the lung neutrophil surge in A/J mice.

### A/J neutrophils have reduced neutrophil extracellular trap release and reactive oxygen species generation early in infection

While neutrophils can kill virus or infected cells, the neutrophil armament contributes heavily to tissue damage particularly if the innate immune response becomes dysregulated [29]. Additionally, the localization of neutrophils in the lung parenchyma is regarded as a hallmark of ARDS and may contribute to the increased pulmonary vascular leakage of A/J mice that we detected late in infection. Here, we sought to determine the relative capacity of neutrophil NET release and ROS generation, two primary mediators of neutrophil-driven lung injury, over the course of influenza infection between strains.

**Fig 3:**
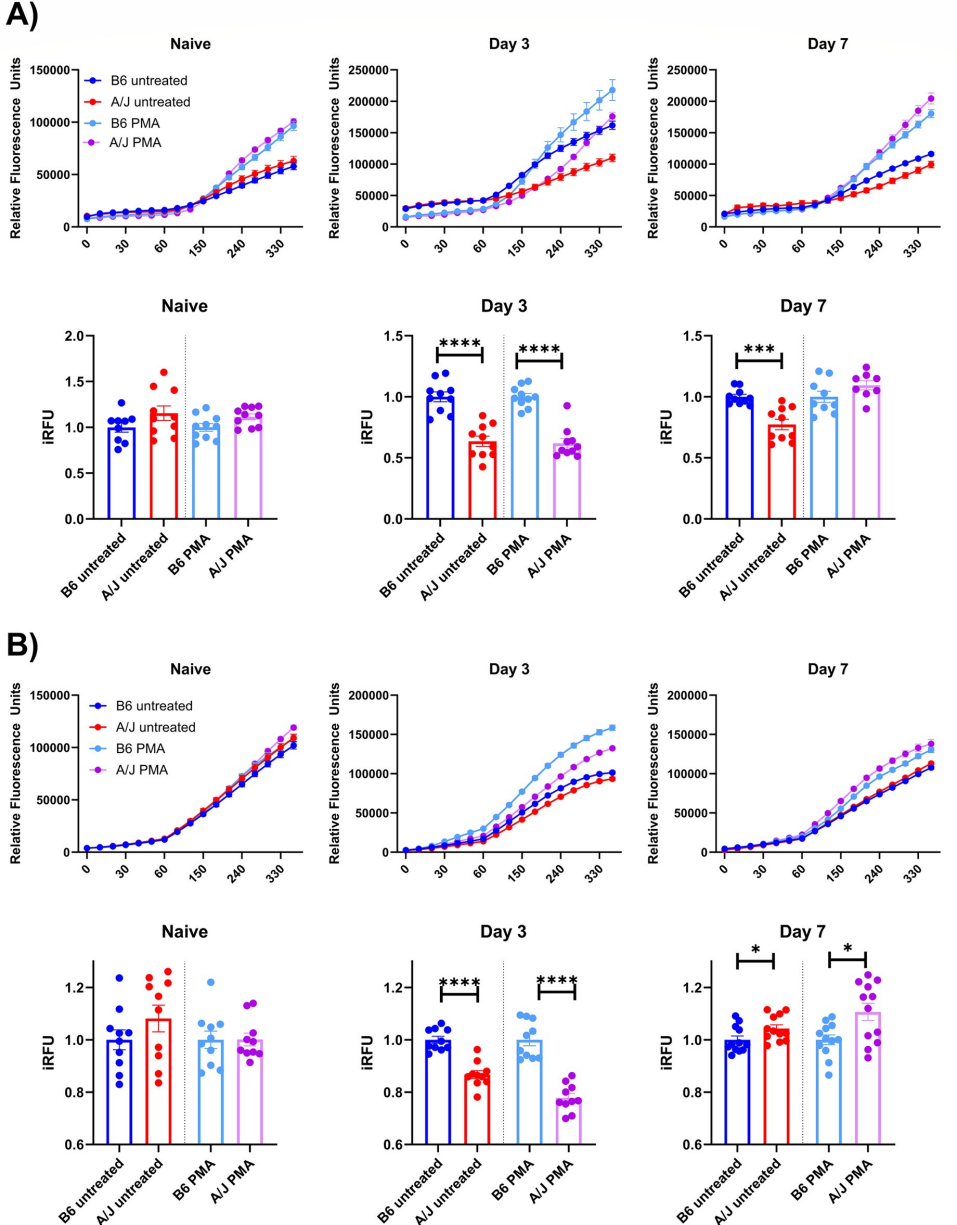
A/J neutrophil have reduced neutrophil extracellular trap release and generate reduced reactive oxygen species compared to their B6 counterparts early in infection but their phenotype shifts late in infection. A) NET release was quantified from bone marrow neutrophils isolated from naïve mice and at days 3 and 7 PI using Sytox Green fluorescent dye assay. For statistical analysis, iRFU was calculated to compare A/J to B6 neutrophils (normalized to an average of 1 for B6) with or without PMA stimulation (n = 10-12/group). B) Intracellular ROS generation was quantified from bone marrow neutrophils isolated from naïve mice and at days 3 and 7 PI using the CM-H_2_DCFDA fluorescent dye. For statistical comparison, iRFU was calculated to compare A/J to B6 neutrophils with or without PMA stimulation. (n = 10-12/group). Data presented are representative of 2 experimental repeats. Statistical analysis by Mann-Whitney U test between A/J and B6 neutrophils within each condition. Significant differences are denoted * p<0.05, *** p<0.001, **** p<0.0001.

To quantify NET release, we utilized a Sytox Green plate-based kinetic read assay to detect dsDNA released from bone marrow neutrophils isolated from uninfected mice and at day 3 and day 7 PI. Isolated neutrophils were treated with vehicle control or phorbol 12-myristate (PMA) in the presence of Sytox Green dye to quantify spontaneous or maximal release of NETs. Our assay indicated that PMA-induced NET release begins after about 60 min and has an inflection point before 240 min, as reported by other groups using this technique to measure NET release in mouse neutrophils [30,31]. To statistically evaluate differences between the two strains, we calculated the indexed relative fluorescence units (iRFU) at 240 min for each group and compared the A/J neutrophils to their B6 counterparts with the same treatment. We found no difference in NET release from naïve neutrophils. At day 3, however, A/J neutrophils released significantly less NETs in both spontaneous and maximal release conditions with roughly 60% of the signal of the B6 neutrophils in either condition (Fig 3A). When assayed at day 7 PI, A/J neutrophils still had reduced spontaneous NET release with roughly 80% of the signal of the B6 neutrophils in the unstimulated condition, however, there was no longer a significant difference with PMA stimulation (Fig 3A).

To quantify ROS generation, we used the CM-H_2_DCFDA fluorescent probe to detect intracellular levels of ROS. Similarly to the NET release assay, we found no difference in naïve neutrophil ROS generation but at day 3 PI, A/J neutrophils had heavily reduced ROS generation compared to B6 neutrophils. By day 7 PI, however, A/J neutrophils produced significantly more ROS than B6 neutrophils in both the vehicle control and PMA-stimulated groups (Fig 3B). These results indicate A/J neutrophil effector functions were strongly diminished early in infection relative to B6 neutrophils at a time when A/J neutrophils were absent from the lungs and the B6 neutrophils were highly recruited. After the induction of airway neutrophilia in A/J mice later in infection, however, the NET release (with PMA stimulation) appears to have equalized with B6 neutrophils and their ROS generation appears to have increased compared to B6 neutrophils. We next sought to investigate other phenotypic changes in neutrophils both during development in the bone marrow compartment as well as after recruitment to the lungs during infection.

### A/J mice have elevated systemic inflammation and evidence of emergency granulopoiesis during infection

**Fig 4:**
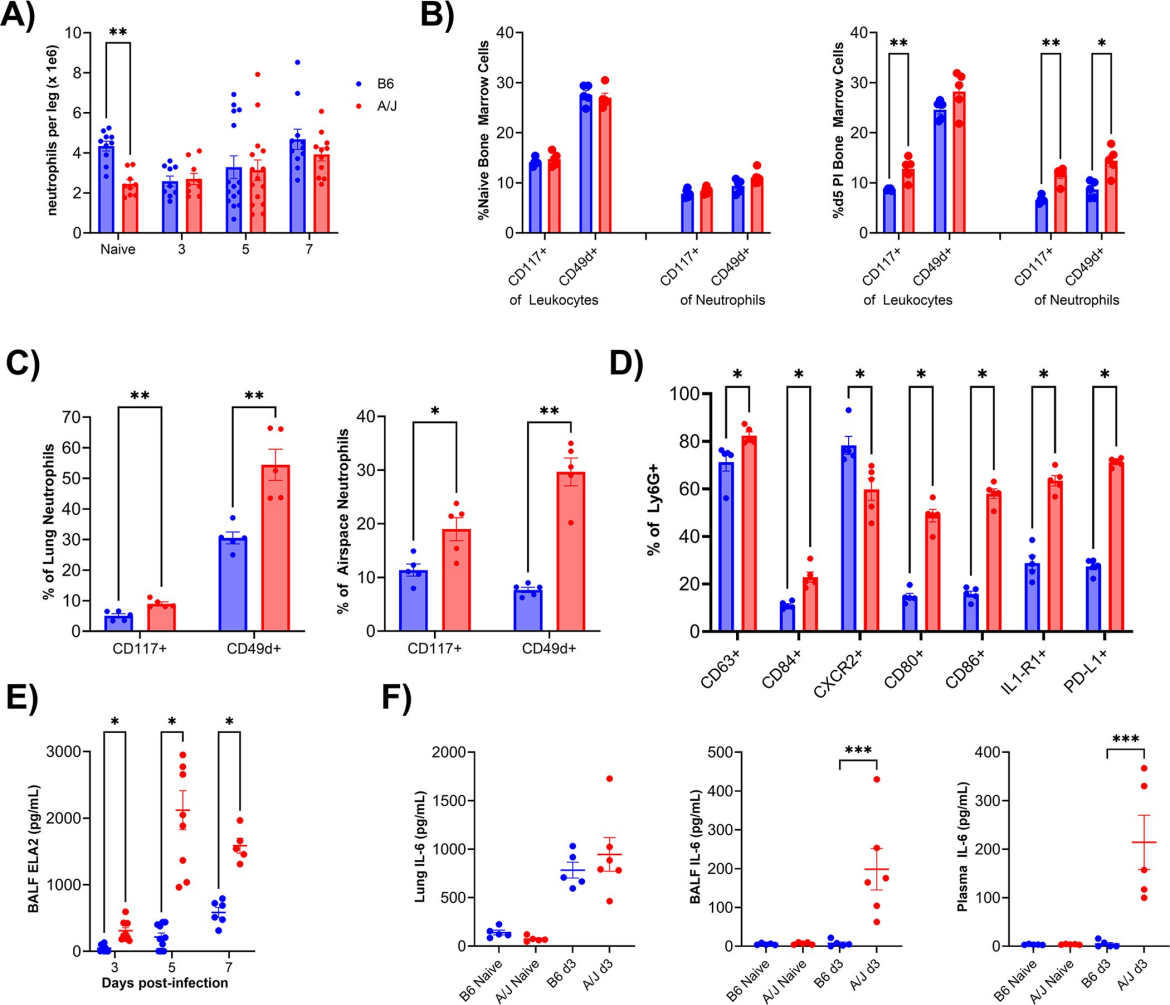
A/J mice have systemic inflammation and a pronounced shift towards immature neutrophils with dysregulated function during infection. A) A/J mice initially have fewer bone marrow neutrophils than B6 mice but after infection there is no longer a difference in numbers recovered. B) Differences in bone marrow neutrophil maturity at naïve state and at day 5 PI were assayed using CD49d and CD117 marker on leukocytes (CD45+) and neutrophils (CD45+, F4/80-, Ly6G+). C) Neutrophil maturity differences were compared in A/J and B6 lung and bronchoalveolar cells at day 7 PI. D) Lung neutrophil cell surface phenotypic markers were compared between B6 and A/J at day 7 PI. E) Neutrophil elastase levels were compared in A/J and B6 BALF at days 3, 5, and 7 PI. F) IL-6 levels were compared in A/J and B6 lung homogenate, BALF, and plasma samples in naïve mice and at day 3 PI. (For A: n = 9-15/group; B-D: n = 5/group; E: n = 6-10/group; F: n = 5-6/group). Data presented are representative of 2 experimental repeats. Statistical analysis by Mann-Whitney U test with two-stage step-up method for multiple comparisons correction, FDR = 0.05 where applicable. Significant differences are denoted * p<0.05, ** p<0.01.

At steady state, we typically found that A/J mice yield fewer bone marrow neutrophils from the femur and tibia (2.5 x 10^6^ cells/leg) compared to age- and sex-matched B6 mice (4.3 x 10^6^ cells/leg) (Fig 4A). During infection, however, the same number of bone marrow neutrophils were recovered from either strain from day 3 PI and after, suggesting a difference in neutrophil granulopoiesis or mobilization between strains (Fig 4A). Emergency granulopoiesis occurs during sepsis and promotes the release of immature neutrophils that have been shown to present with reduced respiratory burst and NET release in humans, mirroring the phenotype of A/J neutrophils early in infection [32,33]. To screen for increased granulopoiesis in the bone marrow we used the markers CD49d, commonly used as a marker of immature neutrophils, and CD117, a marker of neutrophil progenitor cells [34,35]. In the bone marrow of naïve mice, we found no difference in CD49d or CD117 expression in leukocytes (CD45+) or neutrophils (CD45+/Ly6G+), indicating there was no difference in immature neutrophil or progenitor populations at steady state (Fig 4B). At day 5 PI, the time point coinciding with the airway neutrophil surge in A/J mice, we found A/J mice had more leukocytes that were CD117+, as well as more neutrophils that were CD117+ or CD49d+ in their bone marrow, indicating an increased ratio of developing myelocytes and immature neutrophils (Fig 4B). We assessed neutrophil maturity in the lungs at day 7 PI, when neutrophils are abundantly present in both strains of mice. A/J neutrophils from both the lung tissue and airway compartments expressed more CD49d and CD117 than B6 neutrophils at day 7 PI, however (Fig 4C). We found no difference in CD49d and CD117 of lung neutrophils from uninfected A/J and B6 mice, indicating increased recruitment of immature neutrophils to the lungs of A/J mice during infection (S2A Fig).

We further analyzed lung neutrophil cell surface markers at day 7 and found A/J neutrophils had a significant upregulation of markers associated with inflammatory activation, including the type 1 IL-1 receptor (IL-1R1), the primary degranulation marker CD63, and the costimulatory molecules CD80 and CD86. On the other hand, we also found reduced expression of CXCR2, suggesting a hindered response to neutrophilic chemokine gradients, and increased expression of the myeloid-derived suppressor cell-like markers CD84 and PD-L1 (Fig 4D). To determine if these differences rose during infection, we profiled neutrophils from the lungs of uninfected mice. We that A/J lung neutrophils expressed more CD84, IL-1R1, and CD80 but less CD86 than B6 lung neutrophils (S2B Fig). We next assayed these markers from bone marrow neutrophils from uninfected mice to determine if they were constitutively different. We found that A/J bone marrow neutrophils still expressed more CD84, but there were no differences in IL-1R1, CD80, and CD86 compared to B6 neutrophils. Unlike in the lungs, the A/J bone marrow neutrophils also expressed substantially more CXCR2 than their B6 counterparts (S2C Fig). Among our surface markers assayed, bone marrow and lung neutrophils from B6 mice had significant differences in all except CD80 (S2D Fig). In contrast, A/J bone marrow and lung neutrophils were different only in CD63, CD84, and CD86, with the largest difference detected in CD84 (S2E Fig). These data indicate the presence of intrinsic phenotypic differences in bone marrow neutrophils between the two strains that may amplify following recruitment to the lungs. Neutrophil elastase is a major component of neutrophil primary granules and known as a major driver of alveolar damage during ARDS. We found elastase levels throughout infection were higher in A/J BALF supernatants, even at day 3 PI, when B6 neutrophils were most prevalent in the lung tissue while A/J neutrophils were almost absent altogether in the lungs and airspaces (Fig 4E). This suggests A/J neutrophils may have released more tissue-damaging elastase after transmigrating into the airspaces. Interleukin-6 (IL-6) is released during acute inflammation and strongly enhances granulopoiesis [36]. We measured IL-6 from the lung, BALF, and plasma of uninfected mice and at day 3 PI. No differences were found in the uninfected mouse samples. We also found no difference in the lungs of A/J mice compared to B6 mice despite greater viral burden. In the BALF and plasma compartments, however, the A/J mice had significantly higher IL-6 levels indicating both the development of a hyperinflammatory environment in the airspaces and the systemic dissemination of inflammatory signals by day 3 PI (Fig 4F). Taken together, this data suggests that the neutrophil phenotype in the lungs of A/J mice is dysregulated during infection, as they express both activated and immunosuppressive cell-surface markers, and this may be triggered by unhindered viral replication and disseminated inflammation beginning as early as day 3 PI.

### Transfer of B6 bone marrow neutrophils to A/J mouse lungs early in infection promotes viral clearance and reduces systemic inflammation

**Fig 5:**
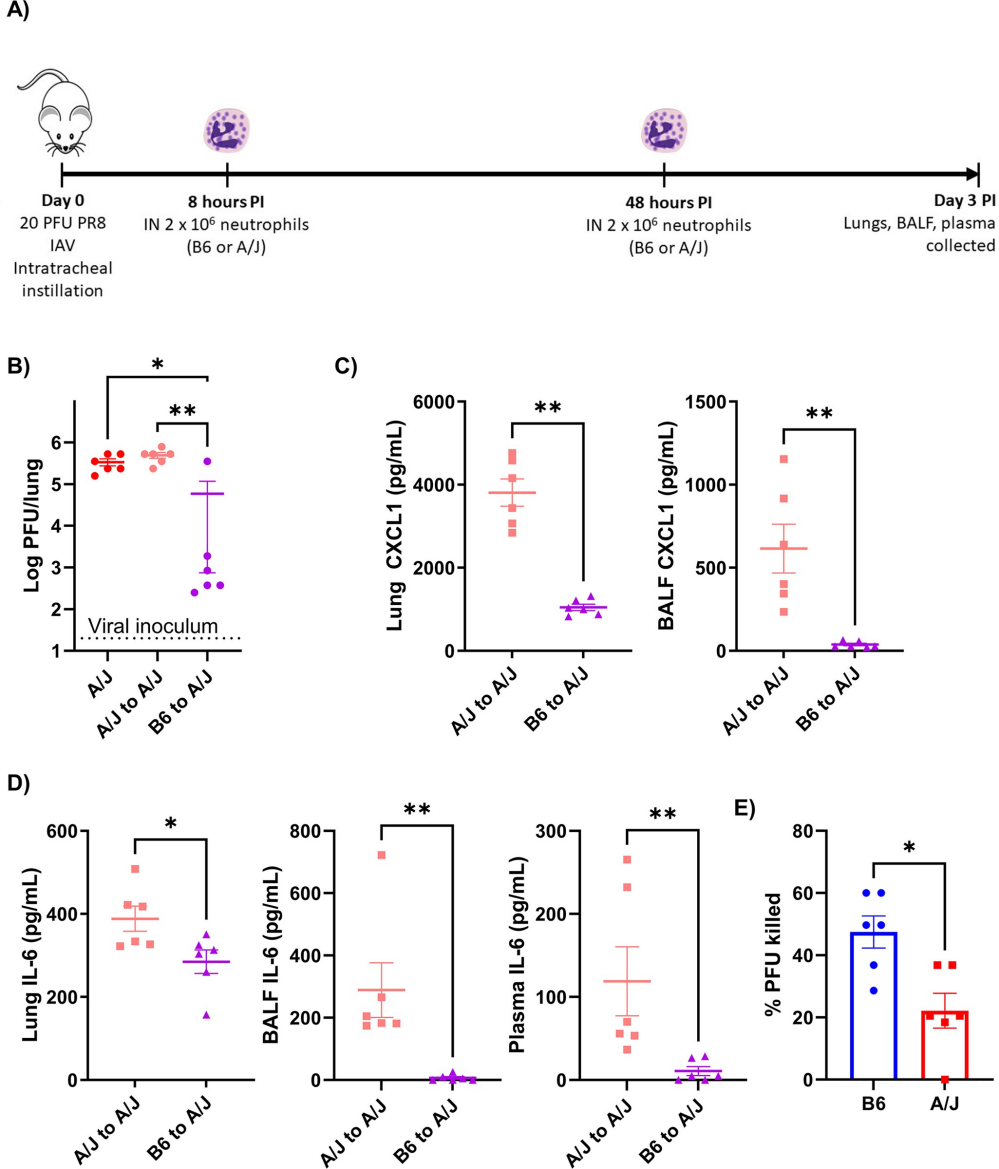
Transfer of B6 but not A/J bone marrow neutrophils into A/J mouse lungs enhances viral clearance and ameliorates inflammation. A) Intranasal transfer of 2 x 10^6^ A/J or B6 bone marrow neutrophils (or vehicle) was performed at 8- and 48-hours post-infection into A/J mice followed by collection of lungs, BALF, and plasma at day 3 PI. B) Lung viral loads were compared between A/J mice receiving vehicle, A/J neutrophils, or B6 neutrophils. C) CXCL1 levels in lung homogenate and BALFsamples were compared at day 3 PI between A/J mice receiving A/J or B6 neutrophils. D) IL-6 levels in lung homogenate, BALF, and plasma samples were compared at day 3 PI between A/J mice receiving A/J or B6 neutrophils. E) The virucidal activity of naïve B6 and A/J bone marrow neutrophils was determined by comparison to cell-free virus controls following 4 hours of virus-neutrophil co-culture. (n = 6/group). Statistical analysis by Mann-Whitney U test with two-stage step-up method for multiple comparisons correction, FDR = 0.05 where applicable or with one-way ANOVA using Tukey’s post-hoc test. Significant differences are denoted * p<0.05, ** p<0.01.

Having found that the B6 mice had a strong presence of neutrophils at day 3 PI that the susceptible A/J mice lacked, we decided to interrogate the functional role of these early responding neutrophils by adoptively transferring neutrophils to infected A/J mice. We isolated bone marrow neutrophils by negative selection from naïve A/J or B6 mice and intranasally transferred 2 x 10^6^ cells to A/J mice at 8- and 48-hours post-infection (Fig 5A). This number of cells corresponds with the difference in neutrophil counts detected in the lungs of A/J and B6 mice at day 3, with a 40-hour delay greater than the reported lifespan of lung neutrophils to avoid an excessive transfer of neutrophils. We found that transferring in B6 neutrophils greatly reduced lung viral loads while transferring in A/J neutrophils had no effect (Fig 5B). Mice that received B6 neutrophils also had significantly reduced CXCL1 levels in the lung and CXCL1 was nearly obviated in the BALF compared to mice that received A/J neutrophils (Fig 5C). Compared to mice that received A/J neutrophils, mice that received B6 neutrophils also had less IL-6 in the lungs and IL-6 was almost completely abrogated in the plasma and BALF (Fig 5D). This data indicates that the early recruitment of neutrophils to the lungs alone is not enough to limit viral replication and inflammation. Instead, there are major functional disparities in A/J and B6 neutrophils that allow only the latter to mediate this benefit.

The presence of neutrophils in B6 mouse influenza infection models have been shown to play critical roles in limiting influenza virus replication and so we hypothesized B6 neutrophils may provide improved viral clearance compared to their A/J counterparts [8,9]. To test this we cocultured naïve bone marrow neutrophils from either strain with virus for 4 hours and compared the resultant viral titers of the supernatants to a cell-free viral control. We found no difference in M gene expression between cell pellets, indicating there was no difference in viral removal either by infection or phagocytosis (S3 Fig). We found that B6 neutrophil virucidal activity was significantly higher compared to A/J neutrophils as they killed nearly half the virus present compared to under a quarter for the A/J neutrophils (Fig 5E). Along with their more rapid response during influenza infection, the enhanced control of virus by B6 neutrophils appears to be crucial in limiting inflammation during infection.

## Discussion

Neutrophil-driven inflammatory injury is regarded as a major driver of ARDS and mortality in influenza infections. In line with previous reports, we have found that A/J mice are highly susceptible to influenza relative to B6 mice, displaying significantly higher mortality and lung viral loads. We found the immune response notably differed between the strains with B6 mice recruiting many neutrophils to lung tissue early in infection while A/J mice had a delayed neutrophil recruitment that localized primarily to the bronchoalveolar spaces. Additionally, A/J mice showed evidence of systemic inflammation by day 3 with increased circulating IL-6, while IL-6 in B6 mice was constrained to the lung tissue. Functional analysis of neutrophils showed A/J neutrophils had greatly reduced NET release and ROS generation compared to B6 neutrophils early in infection. Late in infection, however, PMA-induced NET release was no longer different between strains although spontaneous NET release remained reduced in A/J neutrophils. ROS generation in both spontaneous and PMA-induced conditions was higher in A/J than B6 neutrophils late in infection. Additionally, the surface marker expression of A/J neutrophils suggested a more immature and dysregulated phenotype than B6 neutrophils.

We demonstrate that B6 neutrophils generate an effective antiviral response as transfer of B6 neutrophils to the airspaces of infected A/J mice greatly reduces lung viral titers. We additionally show B6 neutrophils can mitigate systemic inflammation in A/J mice as they preclude the dissemination of IL-6 early in infection. Strikingly, however, we found that the transfer of A/J neutrophils was not able to control either virus or inflammation, demonstrating the presence of major functional differences between neutrophils from the two strains. Emphasizing these differences, we found B6 neutrophils had an improved ability to kill virus *in vitro* compared to A/J neutrophils. Our data indicates that both the early presence of neutrophils in the lungs and their host-specific neutrophil phenotype can affect susceptibility to influenza infection by modulating the early control of viral load and regulating inflammatory tone.

Our results put forth the novel concept that substantial inter-individual differences in neutrophil phenotypes exists, not only under baseline conditions but also during infections. This study and our previous finding that A/J and B6 neutrophils vary significantly in primary granule protein expression and differentially contribute to immunopathology in respiratory coronaviral infections highlight the importance of understanding how interindividual variability in neutrophil phenotypes can create divergent respiratory infection outcomes [37]. Neutrophils have long been recognized as a central therapeutic target in lung injury but the development of neutrophil-specific therapeutics has met little clinical success, with their context-dependent functional heterogeneity playing a large role in this difficulty [38,39]. While neutrophils are closely linked to immunopathology in lung injury, they are also critical for pathogen clearance—although this function is less straightforward in viral infections—and in the regulation and resolution of pulmonary immune responses [39–42]. In humans, neutrophils have been shown to have high interindividual genotypic variability but the extent of their interindividual functional variability is still unclear [43]. Given that human patients present with a spectrum of clinical outcomes in response to the same IAV strain and that neutrophilic inflammatory gene signatures are strongly associated with increased influenza severity in humans, studies are necessary to determine whether interindividual differences in neutrophil functions may govern severity of viral infections [18,44].

Although it is still unclear what drives pathologically excessive neutrophil activation, our findings in the A/J flu model suggest that an initial paucity in inflammatory response and inability to contain virus results in excessive recruitment and activation of neutrophils late in infection. A previous study comparing inflammatory responses in influenza infections in B6 mice with sublethal H1N1 A/Texas/36/91 and lethal PR8 viral infections suggested that viral containment was a major factor driving both neutrophilic inflammation and the lethality of infection, with PR8 able to sustain viral spread within the lungs for multiple days despite the innate immune response [10]. This in turn appeared to promote a “feed-forward inflammatory circuit” leading to broader neutrophil recruitment and a progressively more neutrophilic inflammatory gene signature in the lungs. Neutrophils recruited following the failure of viral containment may further amplify inflammatory pathology and in support of this, attenuating neutrophil numbers with low dose 1A8 antibody treatment was shown to promote survival in severe PR8 influenza infections [10]. Further supporting that an effective initial inflammatory phase is crucial to limit neutrophilic inflammatory pathology, the depletion of neutrophils late in infection had no effect on mortality in flu-infected B6 mice, while depletions starting concurrently with infection increased viral loads, lung damage, and mortality [7,9]. This finding was supported by a later study by our group showing early infection neutrophil depletion substantially reduced survival in young adult mice while late depletion had limited effect [4]. Neutrophils depletions during severe influenza infections in B6 mice have proven deleterious despite the close association of neutrophils with disease severity, and our data suggests this drawback is attributable to neutrophil contributions to viral clearance early in infection that precludes excessive inflammation. Identification of the mechanisms underlying feed-forward inflammatory amplification in neutrophils could provide new therapeutic targets to interrupt the development of neutrophilic pathology. Recent studies have indicated that inflammasome activation by GSDMD is instrumental in the development of neutrophilic pathology in murine influenza models, as GSDMD^-/-^ mice are protected from PR8 influenza and do not show greater benefits with neutrophil depletion as the wild-type mice do [12]. Another study using the HKx31 influenza model found that GSDMD^-/-^ mice had attenuated lung inflammation along with reduced airspace neutrophil infiltration, suggesting that GSDMD may be an underlying trigger of excessive damaging neutrophilic inflammation [45].

Neutrophils in A/J mice had reduced retention in the lungs and reduced virucidal capacity early in infection which contributes to a failure to control viral load. As a result, lung and bronchoalveolar CXCL1 levels as well as circulating IL-6 levels all increased. Transfer of B6 neutrophils was able to mitigate viral load and abrogated the early systemic inflammation seen in A/J mice, whereas transfer of A/J neutrophils had no effect on viral load or inflammation. We observed dysregulated neutrophil phenotypes late in infection of A/J mice compared to B6 mice including reduced maturity, NET release, and CXCR2 expression, and increased respiratory burst capacity, immunosuppressive-like marker expression, costimulatory marker expression, and degranulation. These are all reminiscent of circulating blood neutrophil phenotypes seen in severe sepsis patients in which neutrophils fail to clear pathogens, exhibit immunosuppressive activity, and contribute to tissue pathology [32,33,46–53]. Alongside increased immunosuppressive-like marker expression in A/J neutrophils, we also noted a reduction in CD8+ T cells and NK cells in A/J lungs relative to B6 lungs late in infection. The possible negative role of neutrophil-mediated immunosuppression on viral clearance may be an avenue for future studies. The A/J and B6 flu model allows for the discrimination of specific neutrophil features that develop during dysregulated immune responses. Further comparative functional profiling may reveal beneficial or deleterious responses and help elucidate novel strategies for remediation.

Our data suggests that infection in the lungs may produce systemic signals that regulate developing neutrophil phenotypes as shown by changes in bone marrow neutrophil maturity, NET release, and ROS generation between strains following infection. A recent scRNA-seq study of peripheral blood neutrophils from human sepsis patients has underscored that acute inflammation drives the release of immature neutrophils and biases the expression of neutrophil subsets, most notably differentiated by the presence or absence of CD177 similarly to neutrophils in patients with severe influenza [19,54]. Supporting that systemic factors may affect neutrophil development, a recent study using a mouse cigarette smoke exposure model for chronic obstructive pulmonary disorder (COPD) demonstrated transcriptional and molecular changes in bone marrow neutrophil precursors that were also reflected in murine blood neutrophils and further translated to peripheral blood neutrophils from human COPD patients [55]. Notably, however, certain changes detected in neutrophil subsets were specific to worsened lung function during COPD and not reflected in whole-body inflammatory conditions like sepsis, which suggests that lung-specific signals may circulate and provide an additional layer of influence on neutrophil development [55]. The role of tissue-specific signals in neutrophil development is highlighted by work from Ballesteros *et al*. showing that neutrophils underwent tissue-specific specialization regardless of whether they migrated via circulation or were artificially transferred from the bone marrow directly into tissue [56]. In our model, we found few differences in the phenotypic marker expression of naïve bone marrow neutrophils. In naïve lung neutrophils, however, we found more differences between strains as well as contrasting patterns of change from the bone marrow to the lungs within a strain. It is possible that in addition to intrinsic differences in neutrophil phenotypes, the phenotype of neutrophils from these different strains of mice may further diverge during the process of recruitment and final tissue-specific maturation. Further studies are underway in our lab to determine the balance of systemic and tissue-specific factors in shaping neutrophil function during infection by intranasally transferring bone marrow neutrophils from uninfected, mildly-, or severely-infected mice into mildly- or severely-infected mice and interrogating their function and plasticity following transfer.

We have found that in influenza infection, host-specific neutrophil characteristics appear to strongly influence the course of disease. More effective viral clearance resulted from the early adoptive transfer of B6 neutrophils into A/J mice and additionally limited the dissemination of inflammation. As the first cells recruited to inflamed tissues in overwhelming numbers, neutrophil variability may therefore be a major determinant in the succeeding immune responses and the ultimate outcome of infection. Further studies are necessary to elucidate how neutrophil function differs between individuals and how specific traits may contribute to effective and self-limiting responses to infection.

## Materials and methods

### Ethics statement and mice

C57BL/6J (#000664) and A/J mice (#000646) were obtained from Jackson Laboratory and bred in-house at the Ann Arbor Veterans Affairs Medical Center and provided with food and water *ad libitum*. Mice were housed in specific-pathogen free conditions in microisolator cages. Mice were 14–24 weeks of age and were age and sex-matched for each experiment. All experiments were approved by the Veterans Affairs Institutional Animal Care and Use Committee and were performed in accordance with NIH guidelines.

### Virus and infection

PR8 IAV was obtained from the American Type Culture Collection (ATCC VR-95) and expanded through passage in MDCK cells (ATCC CCL-34) and supernatant collection after 24 hours of infection. Mice were infected with 20 PFU of IAV diluted in sterile phosphate-buffered saline (PBS) in 30μL volume via intratracheal instillation as previously described [57]. Mice were monitored daily for weight and disease symptoms and were euthanized if weight loss exceeded 30% of day 2 post-infection weight or a combination of weight loss and clinical symptoms met humane euthanasia criteria.

### Bronchoalveolar lavage

Mice were euthanized and trachea were intubated with 23-gauge polyethylene tubing attached to a syringe. Lungs were inflated with 1mL PBS with 5mM EDTA. Fluid was then withdrawn after gentle palpation of the lung. For cell differentials, the lungs were lavaged a total of 6 times. Cells were pelleted at 500g for 5min and the 1^st^ mL of BALF supernatant was retained for protein analysis.

### Lung digest

Mouse lung vasculature was perfused with PBS with 5mM EDTA via cardiac puncture of the right ventricle. Lungs were collected and minced before digestion in RPMI medium containing 5% fetal bovine serum (FBS) with 267.8U/mL collagenase IV (Worthington Biochemical CLSS-4) and 50U/mL DNase I (Sigma-Aldrich D4263) at 37°C. Lung digests were repeatedly strained through an 18-gauge needle before being passed through a 70um cell strainer to obtain single cell suspensions. Blood cells were lysed with 2mL ACK buffer for 2min on ice. Following resuspension cells were counted and used for flow cytometry.

### Neutrophil adoptive transfer

To isolate bone marrow neutrophils, mice were euthanized and humerus, femur, and tibia were cleared of skin and muscle tissue in 70% ethanol followed by PBS. Bone marrow was then flushed with 5% FBS RPMI from bones with a 27.5-gauge needle. Bone marrow was then passed through a 20g needle until broken up. Cell suspension was then washed through a 70μm cell strainer. Cells were then layered onto a discontinuous gradient of Hank’s buffered saline solution (HBSS) (Thermo Fisher Scientific 14025) over 55% and 62.5% Percoll (Cytiva 17544501). Cells were then centrifuged at 1000g for 30min. Neutrophils were isolated from the enriched pellet using an EasySep Mouse Neutrophil Enrichment Kit (STEMCELL Technologies 19762) according to manufacturer instructions yielding greater than 95% cell purity (S4A Fig). Isolated neutrophils were washed and transferred intranasally under isoflurane anesthesia at 8- and 48-hours post-infection in 30 μL sterile PBS.

### Flow cytometry

Isolated cells were stained with Zombie Aqua fixable viability dye (BioLegend 423101) and washed in PBS. Fc receptors were blocked with anti-CD16/32 (BioLegend 101320) in 2% FBS-PBS. Cells were then stained with antibodies for desired surface markers. Following staining, cells were washed and fixed in 4% paraformaldehyde buffer (Biolegend 420801). Cells were acquired on an FACSymphony A3 cytometer (BD) and analyzed using FlowJo 10.9.0 (BD). Leukocyte cell counts were obtained by acquiring total cell numbers post lung digest on a Countess II FL (Thermo Fisher Scientific) and calculating against viability and CD45+ rates acquired through flow cytometric analysis. Viable CD45+ cells were assessed for marker expression. The gating strategy for leukocyte cell differential analysis is shown in S5 Fig.

### Antibodies

*AF488*: CD45 (clone: 30-F11, BioLegend), C5L2 (clone: 468705 R&D Systems); *PerCp/Cy5*.*5*: CD45 (clone: 30-F11, BioLegend), CD19 (clone: 6D5, BioLegend), CD49d (clone R1-2, BioLegend); *APC*: F4/80 (clone: BM8, BioLegend), IL-1R1 (clone: JAMA-147, BioLegend); *Alexa Fluor 647*: CD63 (clone: NVG-2, BioLegend), CXCR2 (clone: SA045E1, BioLegend); *APC-Cy7*: CD4 (clone: GK1.5, BioLegend); *PE-Cy7*: CD8a (clone: 53–6.7, BioLegend), TLR4 (clone: SA15-21, BioLegend), CD86 (clone: GL-1, BioLegend), Ly6G (clone: 1A8, BioLegend), CD117 (clone: 2B8, BioLegend); *PE*: CD49b (clone: DX5, BioLegend), Ly6G (clone: 1A8, BioLegend), IL-1R2 (clone: 4E2, BD), MHCII I-A^κ^ (Aßκ) (clone: 10–3.6, BioLegend), MHCII I-A/I-E (clone: M5/114.15.2, BioLegend), CXCR4 (clone: 551696, BD); *BV650*: CD3 (clone: 17A2, BioLegend), PD-L1 (clone: 10F.9G2, BioLegend), CD80 (clone: 16-10A1, BioLegend), CD84 (clone: 1D3/CD84, BD); *BV421*: Ly6G (clone: 1A8, BioLegend), CD34 (clone: MEC14.7, BioLegend).

### CM-H_2_DCFDA reactive oxygen species assay

ROS generation in neutrophils was assayed as previously described [58]. Briefly, bone marrow single cell suspensions were prepared from femur and tibia as described above. After lysing red blood cells, neutrophils were isolated using an EasySep PE positive selection kit (STEMCELL Technologies 17666) according to manufacturer instructions using PE-conjugated anti-Ly6G antibody (clone: 1A8, BioLegend) yielding greater than 98% cell purity (S4B Fig). Neutrophils were plated at a concentration of 2 x 10^6^ cells/mL in black-wall 96-well plates (Corning 3916) in HBSS with 10μM CM-H_2_CDFDA (Thermo Fisher Scientific C6827) for 30min prior to starting the assay. Equal volumes of HBSS or HBSS containing PMA (Thermo Fisher Scientific J63916MCR) were added to the plate to a concentration of 10 μM. Cells were immediately analyzed in a Synergy H1 fluorescence plate reader (BioTek), 493/522nm, and Gen5 3.11 (BioTek) after addition of stimulant. The plates were read every 10min for the first hour then every 30min for 16 hours. Indexed relative fluorescence units (iRFU) were calculated as follows: $iRFU=\frac{maximum\;RFU\;of\;test\;sample}{maximum\;RFU\;of\;reference\;sample}$ where the test sample was from A/J mice and the reference sample was from B6 mice [58,59].

### Sytox green NET release assay

Neutrophils were isolated as described above using the EasySep PE positive selection kit. Neutrophils were plated in black-wall 96-well plates in HBSS with 10 μM Sytox Green (Invitrogen S7020) at a concentration of 2 x 10^6^ cells/mL for 30min prior to starting the assay. An equal volume of HBSS or HBSS containing PMA to a concentration of 10 μM was added to the plate and cells were immediately analyzed as described above.

### ELISA

CXCL1, CXCL2, IL-6, or IgM levels in BALF, lung homogenate, or plasma samples were measured with ELISA kits (CXCL1: R&D systems DY453; CXCL2: R&D systems DY452; IL-6: R&D systems DY406; IgM: Immunology Consultant Labs E90-M) following manufacturer’s instructions. CXCL5, CCL2, CCL3, CCL4, and CCL5 were measured in BALF or lung homogenate samples by LEGENDPlex mouse proinflammatory chemokine panel (Biolegend 740007).

### Neutrophil virucidal assay

Bone marrow neutrophils were isolated as described above using the EasySep PE positive selection kit. Cells were resuspended in viral growth media (DMEM with 1x penicillin/streptomycin, 0.3% bovine serum albumin fraction V, and 25mM HEPES) at a concentration of 5e6 cells/mL in a V-bottom 96-well plate for 30min prior to starting the assay. An equal volume of IAV was added to the neutrophils or to cell-free media to a multiplicity of infection of 1 in the presence of 2.5% mouse serum as a source of complement. Cells and virus were cultured for 4 hours before centrifuging cells. Supernatant was collected and frozen at -80°C before assessing viral load. Viral killing was assessed as the percent reduction of TCID50/mL compared to cell-free controls.

### Viral load assessment

Viral load in tissue and in virus-neutrophil coculture supernatants was assessed using TCID50 in MDCK cell monolayers. Briefly, MDCK cells were grown to 80% confluence in 96-well plates and viral samples were added at dilutions ranging from 5^1^−5^7^ in viral growth media in a 50 μL inoculation volume. After 72 hours of co-culture, cells were fixed, stained with crystal violet, and cytopathic effect was recorded. TCID50/mL was calculated using the modified Spearman-Karber method and converted to PFU/mL with the conversion factor 0.56 TCID50/mL = PFU/mL [60,61].

### Statistics

GraphPad Prism 10.2.3 (GraphPad Software) was used to calculate significant differences between two groups using Mann-Whitney U tests with multiple comparison adjustment by two-stage step-up method of Benjamini, Krieger, and Yekutieli with false discovery rate (FDR) cutoff of 5% where applicable. Two-way ANOVA with Bonferroni’s post-hoc test was used for comparisons between 2 groups across different time points. One-way ANOVA with Tukey’s post hoc test was used to compare experiments with more than 2 groups. Differences in survival were calculated using log-rank test. Data are represented as mean values ± SEM. Significant differences are denoted * p<0.05, ** p<0.01, *** p<0.001, **** p<0.0001.

## Supporting information

S1 Fig Additional neutrophil chemokines in the lung and BALF. CXCL5, CCL2, CCL3, CCL4, and CCL5 chemokines that are involved in inflammatory neutrophil recruitment were surveyed from lung homogenates and BALF of naïve A/J and B6 mice and at days 3, 5, and 7 PI (n = 5/group). Statistical analysis by Mann-Whitney U test with two-stage step-up method for multiple comparison correction, FDR = 0.05. Significant differences are denoted * p<0.05.
(TIF)

S2 Fig Neutrophil phenotypic markers from bone marrow and lungs of uninfected mice. A) Lung neutrophils from naïve A/J and B6 mice were assessed for maturation differences using CD49d and CD117. B) Lung neutrophil cell surface phenotypic markers were compared between naïve B6 and A/J mice. C) Bone marrow neutrophil cell surface phenotypic markers were compared between naïve B6 and A/J mice. D) B6 neutrophil cell surface phenotypic markers were compared between bone marrow and lung compartments. E) A/J neutrophil cell surface phenotypic markers were compared between bone marrow and lung compartments. n = 5/group. Statistical analysis by Mann-Whitney U test with two-stage step-up method for multiple comparison correction, FDR = 0.05. Significant differences are denoted * p<0.05.
(TIF)

S3 Fig Influenza M gene quantification in neutrophil cell pellets. Following virucidal co-culture experiments, RNA was isolated from the cell fraction and M gene was quantified to compare viral titers. n = 5/group. Statistical analysis by Mann-Whitney U test.
(TIF)

S4 Fig Purity of Neutrophil Isolation by negative or positive selection. A) Neutrophils were isolated from bone marrow using negative magnetic selection technique yielding purity >95%. B) Neutrophils were isolated from bone marrow using Ly6G-PE positive magnetic selection yielding purity >98%. Representative flow cytometry gating plots shown.
(TIF)

S5 Fig Cell Differential Gating Strategy. Leukocyte populations were identified in the lungs and BALF from viable singlet cells. Lineage-restricted markers were used for cell identification as follows: B cells (CD45+ F4/80- Ly6G- CD3- CD49b- CD19+), Macrophages (CD45+ F4/80+), Neutrophils (CD45+ F4/80- Ly6G+), NK cells (CD45+, F4/80- Ly6G- CD3- CD49b+), CD4+ T cells (CD45+ F4/80- Ly6G- CD3+ CD4+), and CD8+ T cells (CD45+ F4/80- Ly6G- CD3+ CD8+). Representative flow cytometry gating plots shown.
(TIF)
