# Supplementary figures and images for: Deficient neutrophil responses early in influenza infection promote viral replication and pulmonary inflammation
Source: PLoS Pathog. 2025 Jan 17;21(1):e1012449. doi: 10.1371/journal.ppat.1012449 (PMC11845034; doi:10.1371/journal.ppat.1012449)

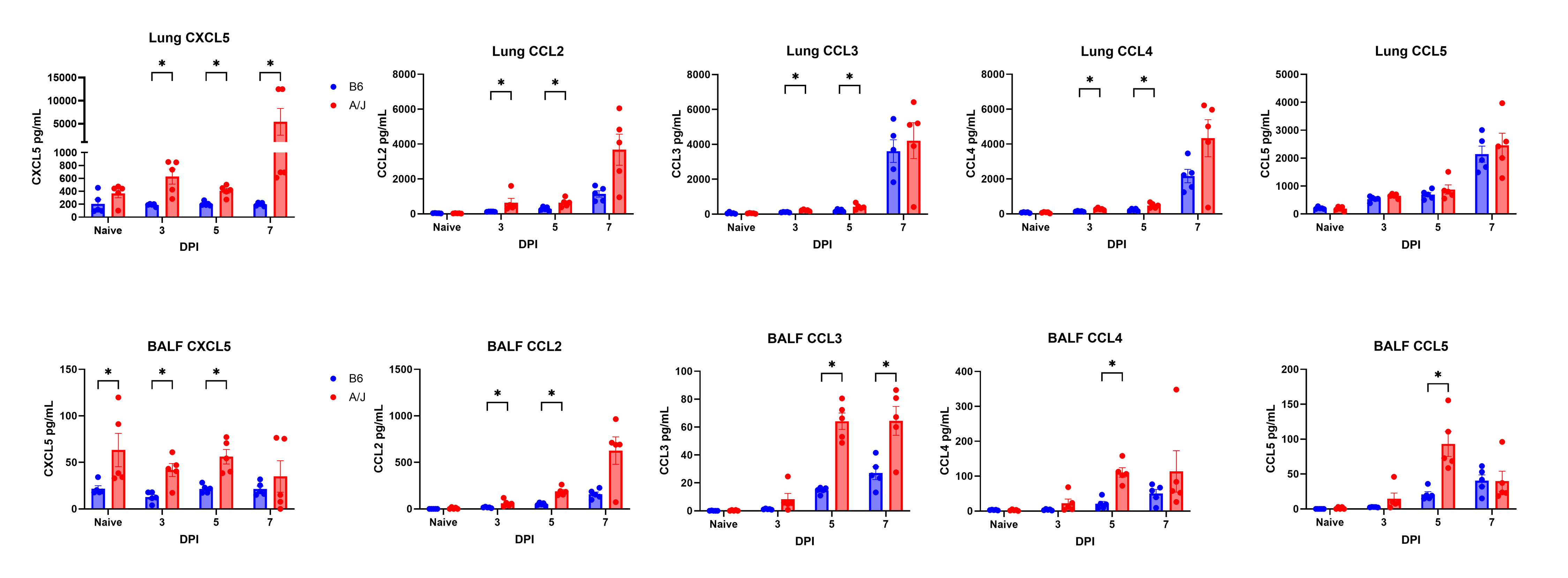

Supplement: S1 Fig — CXCL5, CCL2, CCL3, CCL4, and CCL5 chemokines that are involved in inflammatory neutrophil recruitment were surveyed from lung homogenates and BALF of naïve A/J and B6 mice and at days 3, 5, and 7 PI (n = 5/group). Statistical analysis by Mann-Whitney U test with two-stage step-up method for multiple comparison correction, FDR = 0.05. Significant differences are denoted * p<0.05. (TIF) [file ppat.1012449.s001.tif]

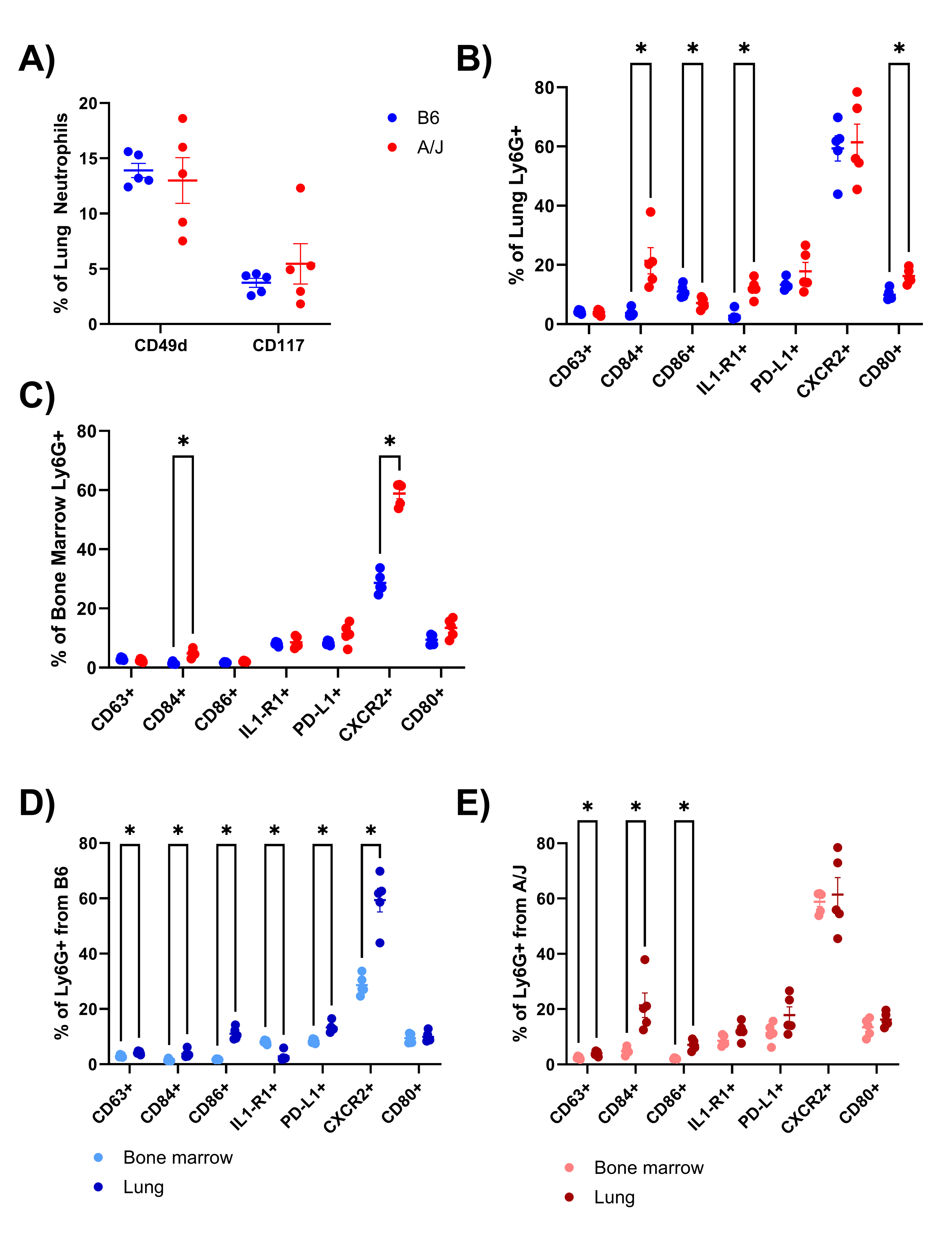

Supplement: S2 Fig — A) Lung neutrophils from naïve A/J and B6 mice were assessed for maturation differences using CD49d and CD117. B) Lung neutrophil cell surface phenotypic markers were compared between naïve B6 and A/J mice. C) Bone marrow neutrophil cell surface phenotypic markers were compared between naïve B6 and A/J mice. D) B6 neutrophil cell surface phenotypic markers were compared between bone marrow and lung compartments. E) A/J neutrophil cell surface phenotypic markers were compared between bone marrow and lung compartments. n = 5/group. Statistical analysis by Mann-Whitney U test with two-stage step-up method for multiple comparison correction, FDR = 0.05. Significant differences are denoted * p<0.05. (TIF) [file ppat.1012449.s002.tif]

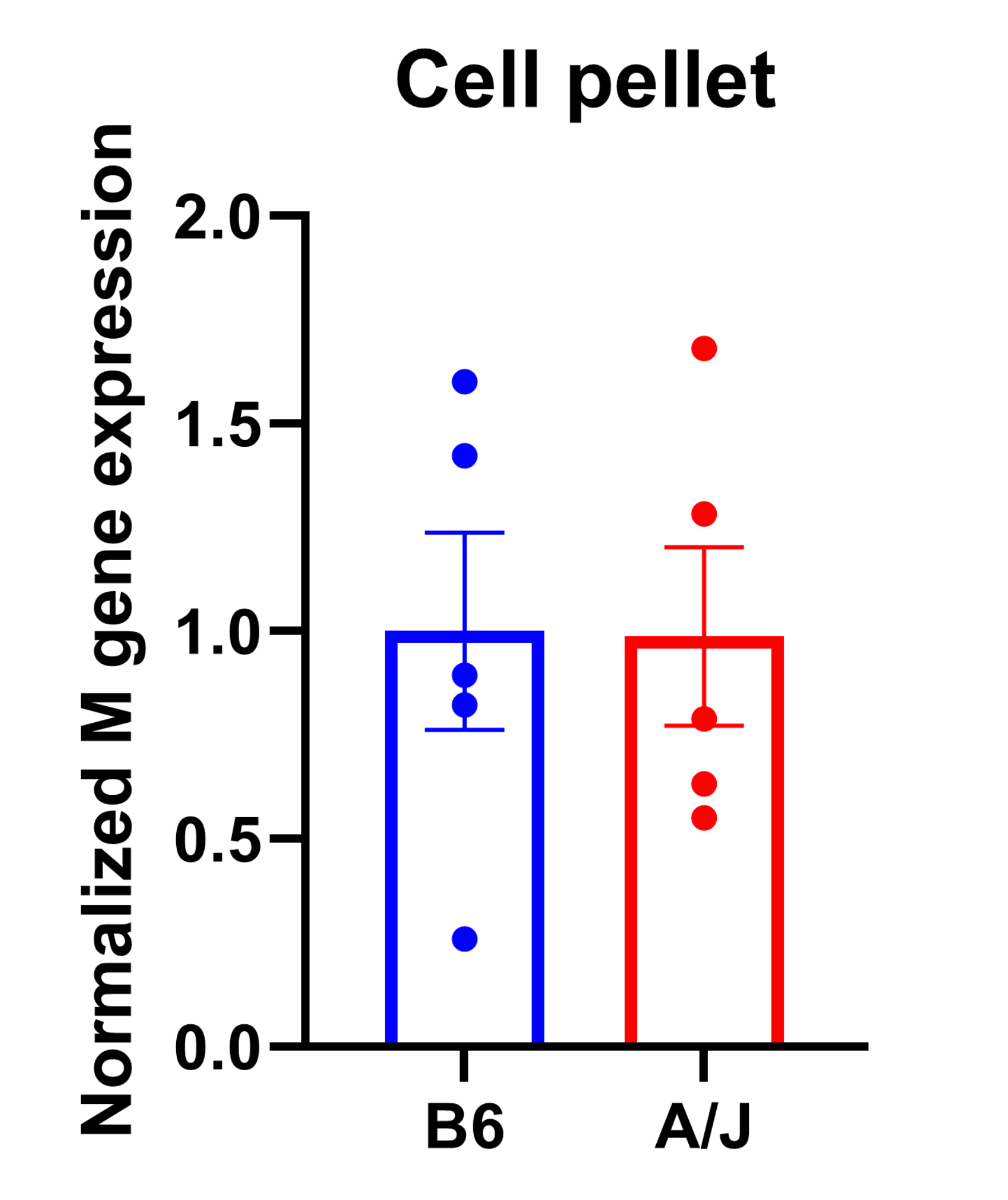

Supplement: S3 Fig — Following virucidal co-culture experiments, RNA was isolated from the cell fraction and M gene was quantified to compare viral titers. n = 5/group. Statistical analysis by Mann-Whitney U test. (TIF) [file ppat.1012449.s003.tif]

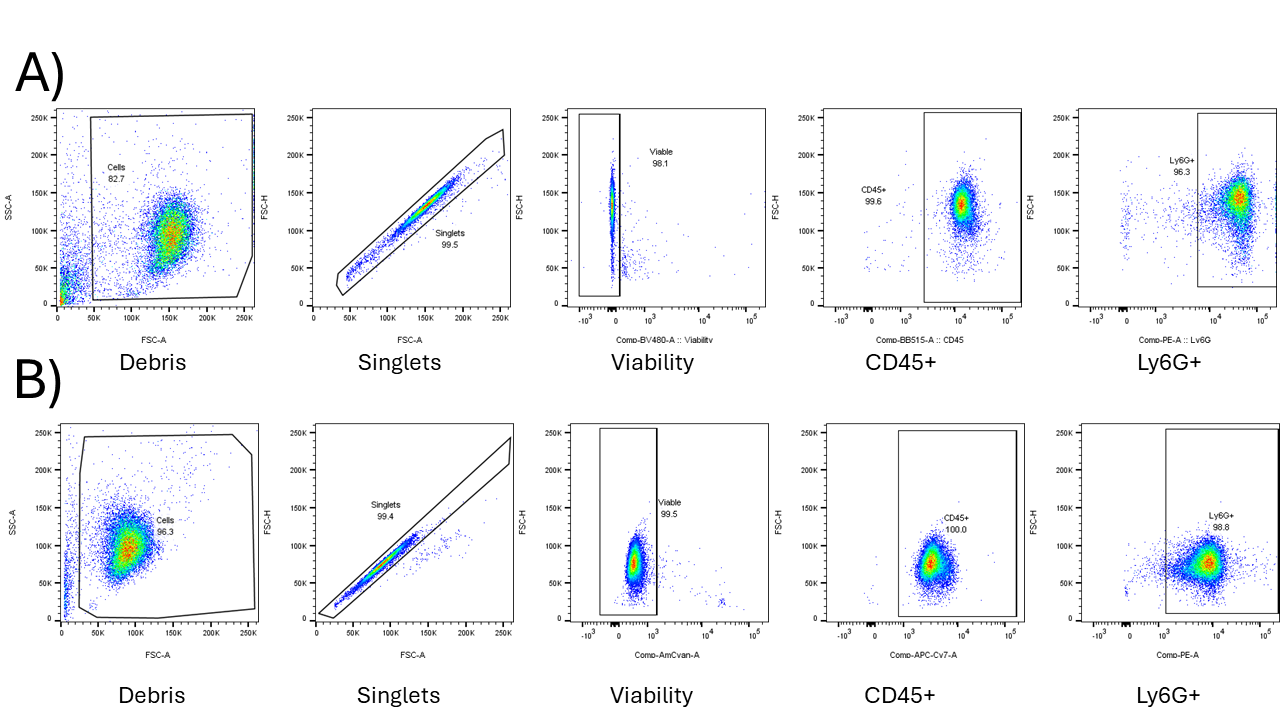

Supplement: S4 Fig — A) Neutrophils were isolated from bone marrow using negative magnetic selection technique yielding purity >95%. B) Neutrophils were isolated from bone marrow using Ly6G-PE positive magnetic selection yielding purity >98%. Representative flow cytometry gating plots shown. (TIF) [file ppat.1012449.s004.tif]

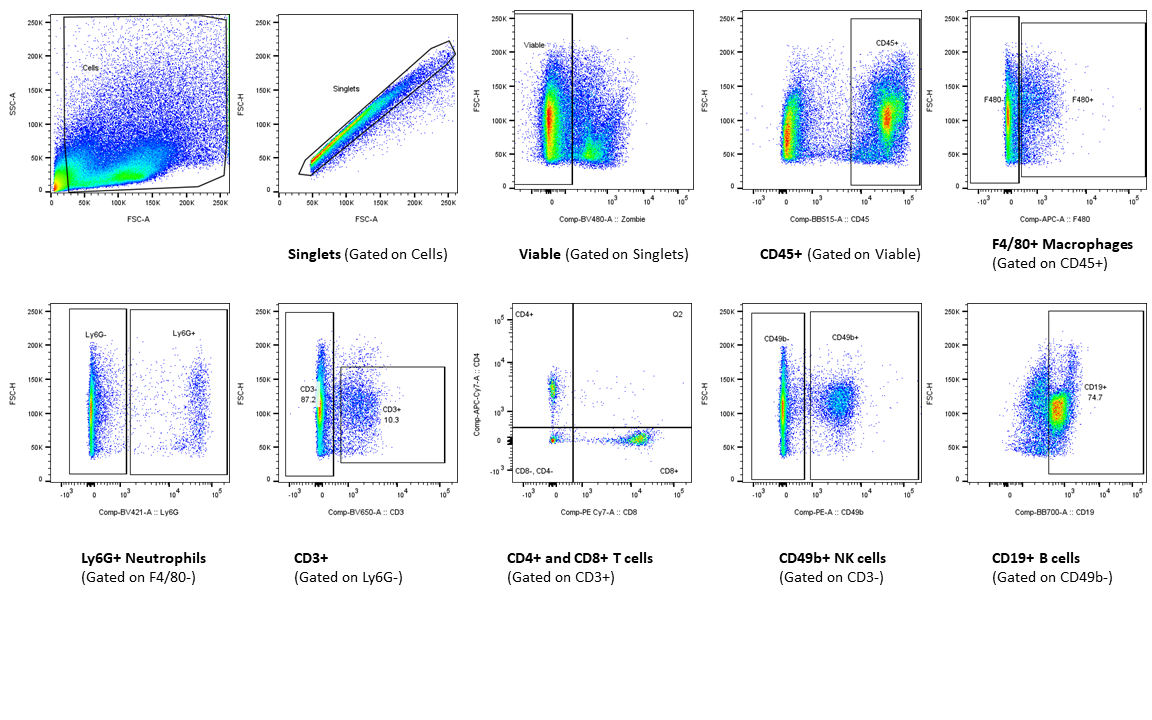

Supplement: S5 Fig — Leukocyte populations were identified in the lungs and BALF from viable singlet cells. Lineage-restricted markers were used for cell identification as follows: B cells (CD45+ F4/80- Ly6G- CD3- CD49b- CD19+), Macrophages (CD45+ F4/80+), Neutrophils (CD45+ F4/80- Ly6G+), NK cells (CD45+, F4/80- Ly6G- CD3- CD49b+), CD4+ T cells (CD45+ F4/80- Ly6G- CD3+ CD4+), and CD8+ T cells (CD45+ F4/80- Ly6G- CD3+ CD8+). Representative flow cytometry gating plots shown. (TIF) [file ppat.1012449.s005.tif]
